# Temporal Variability of Human Vaginal Bacteria and Relationship with Bacterial Vaginosis

**DOI:** 10.1371/journal.pone.0010197

**Published:** 2010-04-15

**Authors:** Sujatha Srinivasan, Congzhou Liu, Caroline M. Mitchell, Tina L. Fiedler, Katherine K. Thomas, Kathy J. Agnew, Jeanne M. Marrazzo, David N. Fredricks

**Affiliations:** 1 Vaccine & Infectious Disease Institute, Fred Hutchinson Cancer Research Center, Seattle, Washington, United States of America; 2 Department of Obstetrics & Gynecology, University of Washington, Seattle, Washington, United States of America; 3 Center for AIDS and STDs, University of Washington, Seattle, Washington, United States of America; 4 Department of Medicine, University of Washington, Seattle, Washington, United States of America; 5 Department of Microbiology, University of Washington, Seattle, Washington, United States of America; Columbia University, United States of America

## Abstract

**Background:**

Little is known about short-term bacterial fluctuations in the human vagina. This study used PCR to assess the variability in concentrations of key vaginal bacteria in healthy women and the immediate response to antibiotic treatment in women with bacterial vaginosis (BV).

**Methodology/Principal Findings:**

Twenty-two women assessed for BV using Amsel's criteria were evaluated daily for 7 or 14 days, then at 2, 3 and 4 weeks, using a panel of 11 bacterium-specific quantitative PCR assays. Participants with BV were treated with 5 days of intravaginal metronidazole. Participants without BV had vaginal biotas dominated by lactobacilli, whose levels fluctuated with menses. With onset of menstruation, quantities of *Lactobacillus jensenii* and *Lactobacillus crispatus* decreased and were found to be inversely related to *Gardnerella vaginalis* concentrations (p<0.001). Women with BV had a variety of fastidious bacteria whose concentrations dropped below detection thresholds 1–5 days after starting metronidazole. Recurrent BV was characterized by initial profound decreases of BV-associated bacteria after treatment followed by subsequent increases at relapse.

**Conclusions/Significance:**

The microbiota of the human vagina can be highly dynamic. Healthy women are colonized with *Lactobacillus* species, but levels can change dramatically over a month. Marked increases in *G. vaginalis* were observed during menses. Participants with BV have diverse communities of fastidious bacteria that are depleted by vaginal metronidazole therapy. Women with recurrent BV initially respond to antibiotic treatment with steep declines in bacterial concentrations, but these bacteria later reemerge, suggesting that antibiotic resistance in these bacteria is not an important factor mediating BV recurrence.

## Introduction

The human vagina hosts communities of microbes that can impact the health of women and their neonates. Bacterial vaginosis (BV) is a common condition, affecting ∼29% of reproductive age women in the USA [1] and is associated with an increase in the risk for pre-term birth [2], HIV-1 acquisition [3] and pelvic inflammatory disease [4]. The pathogenesis of BV is poorly understood. BV is associated with loss of vaginal lactobacilli such as *Lactobacillus crispatus* and *Lactobacillus jensenii*, and acquisition of complex communities of anaerobic bacteria. Although the vaginal bacterial biota has been intensively investigated using cultivation (reviewed in [5], [6]) and molecular approaches (reviewed in [7]), few studies have measured short-term fluctuations in the vaginal microbiota using cultivation-independent methods. Persistent or relapsing BV is a common problem with relapse rates greater than 50% within 6 months of treatment [8]. Taxon-directed quantitative PCR (qPCR) has been used to measure levels of key vaginal bacteria and how they change after antibiotic treatment in women with cured and persistent BV [9], but that study did not measure immediate responses to antibiotic treatment. It is unknown whether bacterial eradication was ever achieved, particularly in women with persistent BV. This raises the question of whether the bacteria are non-responsive to antibiotic treatment in women with recurrent BV, or whether the bacteria are eradicated but return with re-inoculation. If the latter situation is most relevant, when and how women with recurrent BV are re-infected is not clear. Longitudinal studies with frequent sampling immediately after antibiotic administration are critical in addressing these questions. Furthermore, the degree of stability of the vaginal bacterial community in women without BV in not well understood. How dynamic is the vaginal microbiota and are BV-associated bacteria detected in women without BV at low concentrations? We sought to address these questions by evaluating key vaginal bacterial levels from samples collected longitudinally from healthy women and women with BV using a panel of taxon-specific bacterial qPCR assays.

## Materials and Methods

### Study population, clinical evaluation and sample collection

Thirty-three subjects were recruited for this longitudinal study from the Public Health - Seattle and King County Sexually Transmitted Diseases Clinic (STD clinic) between September 2006 and May 2009. The study was approved by the Institutional Review Board at the Fred Hutchinson Cancer Research Center. Written informed consent was obtained from all study participants. Participants were evaluated in clinic on Day 0 (recruitment), and returned to clinic for examination at 1 month (25–49 days; Median = 28). All participants were asked to self-collect vaginal swabs daily for 7 or 14 days, then at 2 weeks and 3 weeks. Self-collected swabs were mailed in on the day of collection at room temperature and held at −80°C before processing. In twelve additional participants, swabs were also collected at 2, 3 and 4 months after study entry. If participants were diagnosed with BV at 4 months, they were offered to re-enroll in the study, facilitating the observation of women with recurrent BV.

At clinic visits, participants were interviewed regarding medical history and sexual behaviors, and underwent a standard physical examination including a pelvic exam with speculum for collection of samples. At the enrollment visit, swabs of vaginal fluid samples were collected for Gram staining, pH, saline microscopy and KOH preparation. Samples for DNA extraction and PCR were obtained using polyurethane foam swabs (Epicenter Biotechnologies, Madison, WI) that were brushed against the lateral vaginal wall, re-sheathed and frozen immediately at −20°C and subsequently held at −80°C. At clinic visits, BV was diagnosed using Amsel's clinical criteria [10] and vaginal fluid smears were collected for Gram stains by the Nugent method [11] to confirm the presence of BV. Subjects with BV, diagnosed using Amsel's clinical criteria, were treated with 5 g 0.75% intravaginal metronidazole gel used each night for 5 days. Testing for STDs and other vaginal infections was performed as previously described [9]. A daily diary was maintained by the participants where they recorded menstruation, medication use and symptoms.

### DNA extraction and quality control

DNA was extracted using the Ultra Clean Soil DNA Kit (Mobio, Carlsbad, CA) [12], eluted in 150 µl buffer and diluted 1∶1 in 1 mM Tris, 0.1 mM EDTA buffer. Two µl of the diluted DNA was used in each qPCR assay. Sham digests from swabs without human contact assessed contamination from reagents or collection swabs. Human 18S rRNA gene qPCR measured human DNA levels verifying that the swab contacted a human tissue surface and documenting successful extraction [13]. All DNA samples were tested for PCR inhibition using a sensitive qPCR approach targeting a segment of exogenously added jellyfish DNA at a concentration suitable for detection of low levels of inhibitors [13]. Inhibition was defined as a delay in the threshold cycle by ≥2 cycles compared to the no-DNA-template controls.

### Quantitative PCR

Eleven qPCR assays targeting key vaginal bacteria associated with health or BV were applied to the samples. Assays for the detection of *Gardnerella vaginalis*, *Lactobacillus crispatus*, *Megasphaera*-like bacterium (type 1 & type 2), *Atopobium vaginae*, *Leptotrichia* and *Sneathia* species (single assay) and three *Clostridiales* Order bacteria designated as bacterial vaginosis associated bacterium 1 (BVAB1), BVAB2 and BVAB3 were applied as described previously [9]. We developed three additional assays targeting *Lactobacillus jensenii*, *Lactobacillus iners*, and a combined assay for *Mobiluncus curtisii* and *Mobiluncus mulieris* using a probe-based assay format [9] with 16S rRNA gene-specific primers, and a taxon-directed hydrolysis probe. Core reagents were from Applied Biosystems (Carlsbad, CA) and master mixes contained buffer A (1 mM), deoxynucleotide triphosphates (1 mM), magnesium (3 mM), AmpErase uracil-N-glycosylase (0.05 U) and AmpliTaq Gold polymerase (1–1.5 U) per reaction. Primers were added at 0.8 µM per reaction (*Mobiluncus* assay-1.2 µM forward primer) and final probe concentration was 150 µM. Assays underwent 45 cycles of amplification on the Eppendorf Mastercycler *realplex* Thermal cycler (Hamburg, Germany). Specificity and sensitivity were defined as described previously [9]. Plasmid standards were run in duplicate from 10^6^ to 2.5 copies, and values are reported as 16S rRNA gene copies/swab. Assay details are provided in Table 1.

**Table 1:** Primer and probe sequences used in the quantitative PCR assays developed in this study.

PCR Assay	PCR Conditions	Amplicon Size	Primer/Probe	Primer/Probe Sequence
***Mobiluncus*** ** spp.**	60°C annealing, 39 s	219 bp	805F_Mob	5′-CCACGCTGTAAACGTTGGGAA-3′
	72°C extension, 30 s		1023R_Mob_curt	5′-TGGCCCATCTCTGGAACCA-3′
			1024R_Mob_mul	5′-CCACACCATCTCTGGCATG-3′
			Mob_838-861	5′-FAM-ATGCTATCCTGTGTTTCTGCGCCGTAG-TAMRA-3′
***Lactobacillus iners***	55°C annealing, 39 s	76 bp	165F_Liners	5′-GATGCTAATACCGGATAAYAACAGAT-3′
	72°C extension, 30 s		241R_Liners	5′-CACCGCAGGTCCATCCAAGA-3′
			Liners_186-213	5′-FAM-TGCCTATCAACTGTTTAAAAGATGGTTCT-TAMRA-3′
***Lactobacillus jensenii***	61°C annealing, 30 s	69 bp	988_Ljens	5′-GTCTTGACATCCTTTGACCAC-3′
	72°C extension, 30 s		1057R_Ljens	5′-CATGCACCACCTGTCTCTTT-3′
			Ljens_1009-1028	5′-FAM-CTAAGAGATTAGGTTTTCCC-MGBNFQ-3′

### Statistics

To determine differences in DNA recovery in clinic-collected versus self-collected swabs, we used the Wilcoxon signed rank test to compare paired medians of the quantities of human 18S rRNA gene copies, *G. vaginalis, L. jensenii and L. crispatus* from 14 women without BV, obtained within one day of each other. To examine apparent patterns in levels of bacteria relative to menstruation, mean differences in log_10_ quantities of bacteria during menstruation were estimated using a linear mixed model, adjusting for treatment and with subjects as random effects to account for correlation in repeated measurements on each subject. Bacterial levels, when not detected, were considered to be half the detection limit.

## Results

### Subject characteristics

Of the 33 participants enrolled in the study, 22 collected at least 10 swabs and were included in the study analysis. Eight participants were diagnosed with BV by Amsel's clinical criteria at their initial visit, and 14 participants were not diagnosed with BV. Subject characteristics are noted in Table 2. A total of 355 swabs were processed, including 192 swabs from healthy women and 163 swabs from women with BV.

**Table 2:** Demographic data of the women enrolled in the longitudinal study.

Participant characteristic	
Mean Age	31.3
Age Range	22–42
Median Age	30.5
	1 **N (%)**
**Race**	
White	13 (59.1%)
African American^2^	5 (22.7%)
Asian	2 (9.1%)
Did not answer	2 (9.1%)
Douching during the sampling period^3^	1 (4.2%)
Other vaginal medication^3^	3 (12.5%)
Vaginal product (not medication)^3^	5 (20.8%)
Sexual activity^3^	18 (75%)

1 N indicates number of participants.

2 Four of five participants reported another race.

3 Data missing for 2 participants, hence the number of women included in the calculation is 20, corresponding with 24 menstrual cycles captured. The denominator used for the calculations is 24.

### Comparison of self-collected and clinic-collected swabs

There was no significant difference in paired median levels of human 18S rRNA gene copies, *G. vaginalis* or *L. jensenii* levels (p>0.05) between clinic- and home-collected swabs. Median levels of *L. crispatus* were higher in the clinic obtained swabs (p = 0.02). These data suggest that human and bacterial DNA levels were largely similar in clinic- and home-collected swabs.

### Variability in vaginal bacterial levels in women without BV

**Figure 1:**
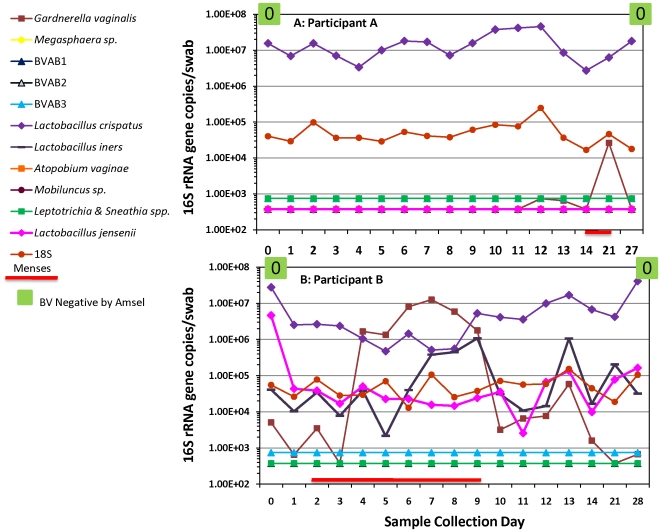
Variability of vaginal bacteria in women without BV. The line graphs show the changes in vaginal bacteria measured as 16S rRNA gene copies per swab in a participant exhibiting a stable bacterial biota (A) and another with dynamic patterns (B). The red line indicates menses. Clinical diagnosis was performed using Amsel's criteria (green box in upper graph indicating negative for BV). Gram staining using the Nugent criteria (the score is indicated as a number from 0–10 in the box) was also performed. A score of 0–3 indicates negative for BV, 4–6 reflects intermediate BV flora and 7–10 denotes positive for BV. Levels of the human 18S rRNA gene (red circles) were used to assess the amount of vaginal fluid loaded on each swab as reflected by human cell content. BVAB denotes bacterial vaginosis associated bacterium.

Taxon-directed bacterial qPCR assays demonstrated that healthy women were colonized with lactobacilli and typically did not harbor BV-associated bacteria with the exception of *G. vaginalis*. However, daily changes in bacterial concentrations were common. Quantities of human 18S rRNA gene copies by qPCR were relatively constant showing consistent loading of human cells on the swabs with good stability of DNA and fluctuations in DNA levels were not due to sample quality (Figure 1). An example of a relatively stable vaginal microbiota is provided in Figure 1A, where participant A is negative for BV at study entry. The participant was colonized with *L. crispatus* and quantities remained relatively stable throughout the period sampled. An increase of >1-log *G. vaginalis* is observed at 3 weeks during menses. In contrast, as demonstrated in Figure 1B, concentrations of *Lactobacillus* species can fluctuate in participants without BV. A 4-log increase in *G. vaginalis* levels was observed along with an increase in *L. iners* coincident with menses, and quantities decreased without intervention at the end of menstruation.

### Variability of Gardnerella vaginalis during menses

**Figure 2:**
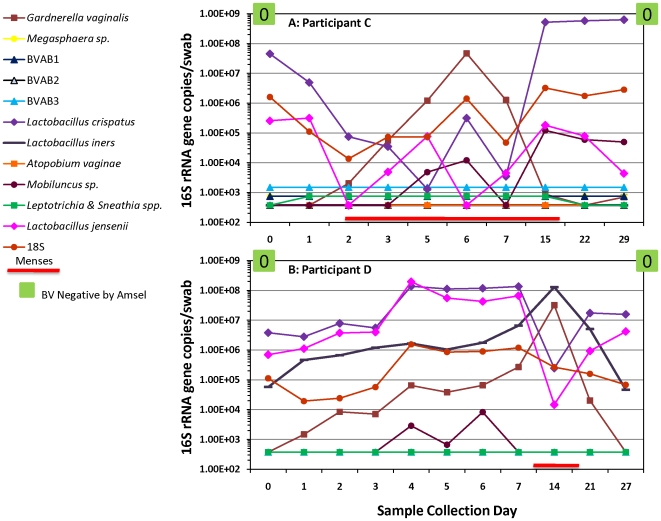
Variability of *Gardnerella vaginalis* during menses. The line graphs show increases in *G. vaginalis* levels during menses while concentrations of *L. crispatus* and *L. jensenii* decreased. *G. vaginalis* quantities declined below detection thresholds post-menses with simultaneous increases in *L. jensenii* and *L. crispatus* levels. Participant C (Figure 2A) was not colonized with *L. iners* while Participant D (Figure 2B) was. Levels of *L. iners* were observed to increase with *G. vaginalis* concentrations and subsequently declined post-menses. Both participants were negative for BV by Amsel's criteria (green boxes) and had Nugent scores of 0 at the entry and follow up visits.

An increase in *G. vaginalis* levels associated with menses, accompanied by decreased quantities of *L. jensenii* and *L. crispatus* were observed (Figures 1 & 2). At the end of menstruation, *G. vaginalis* quantities decreased to below detection limits (Figures 1 & 2) and *L. iners* concentrations declined as well (Figures 1B & 2B); simultaneous increases in quantities of *L. crispatus* and *L. jensenii* were observed. On average, quantities of *L. crispatus* were found to be lower during menstruation by 0.6 logs (p = 0.001) while *G. vaginalis* quantities were found to be higher by 1.38 logs after adjusting for sampling during treatment (Table 3). Quantities of *L. jensenii* and *L. crispatus* were both found to be inversely related to *G. vaginalis*; *L. jensenii* was lower by 0.19 logs per log_10_ increase in *G. vaginalis* (p<0.001) and *L. crispatus* by 0.17 logs (p<0.001).

**Table 3:** Differences in levels of bacteria by qPCR during menstruation.

Bacterium	mean log_10_ adjusted difference (95% CI), p-value* during menstruation
*Lactobacillus crispatus*	−0.60 (−0.94, −0.25), p = 0.001
*Lactobacillus jensenii*	−0.39 (−0.79, 0.01), p = 0.06
*Lactobacillus iners*	0.10 (−0.23, 0.43), p = 0.56
*Gardnerella vaginalis*	1.38 (0.83, 1.93), p<0.001

Data shown for 16 subjects who reported days with and without menses during the study period.

Mean log_10_ adjusted change, p-values, and 95% CIs (confidence intervals) estimated using a linear mixed model with participants as random effects and adjusted for treatment. We tested those patterns that appeared interesting upon examination of the data; thus, these p-values and 95% CIs arise from exploratory analyses and serve as a basis for future hypothesis-driven research.

### Eradication of BV-associated bacteria post antibiotic treatment

**Figure 3:**
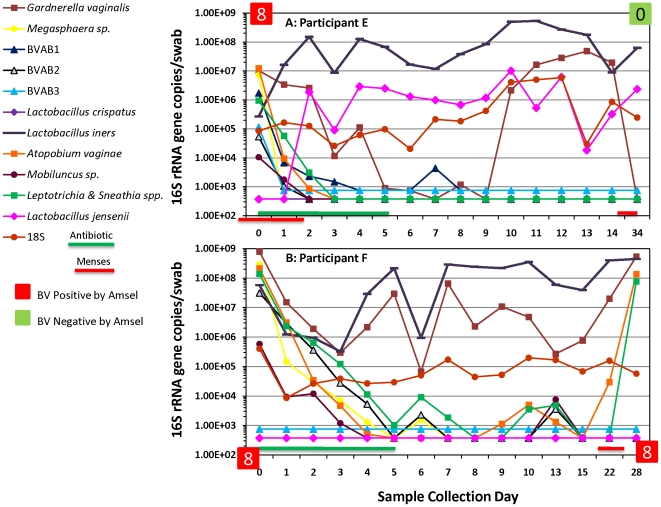
Eradication of BV bacteria post-antibiotic treatment. Participant E (Figure 3A) and Participant F (Figure 3B) are two women with BV by Amsel criteria (red boxes) and by Nugent criteria (Score = 8) at study entry. There was a difference in the rate of decrease of BV bacteria post-metronidazole therapy. Participant E was cured of BV at the 1 month visit as determined by Amsel's (green box) and Nugent's (Score = 0) criteria. Participant F was re-colonized with *A. vaginae*, *Leptotrichia* & *Sneathia* spp. and *G. vaginalis*; BV was diagnosed at that time. The green line at the bottom of the graph shows days of metronidazole treatment and the red line denotes menses.

With the exception of *G. vaginalis*, quantities of BV-associated bacteria decreased several logs and dropped below detection thresholds between Days 1 and 5 (Median = 3.5 days) of metronidazole treatment (Figure 3) in women with BV. We show data from two representative subjects.

Participant E had high levels of BV-associated bacteria at diagnosis which declined below detection thresholds (except *G. vaginalis*), while concentrations of *L. jensenii* increased 3.5-logs. High concentrations of *L. iners* were observed throughout the period sampled.

In contrast, the rate of decrease of BV-associated bacteria in Participant F was slower. Participant F maintained moderate to high *G. vaginalis* levels throughout the period sampled. There were transient increases in concentrations of some BV-associated bacteria after eradication on Day 5, but they resolved by Day 15. However, an elevation of *G. vaginalis*, *A. vaginae* and *Leptotrichia/Sneathia* spp. quantities were observed during menses. Participant F was subsequently diagnosed with BV at her 1-month clinic visit. Participant F was never colonized with *L. crispatus* or *L. jensenii* throughout the period sampled.

### Variability of bacteria in women with recurrent BV

**Figure 4:**
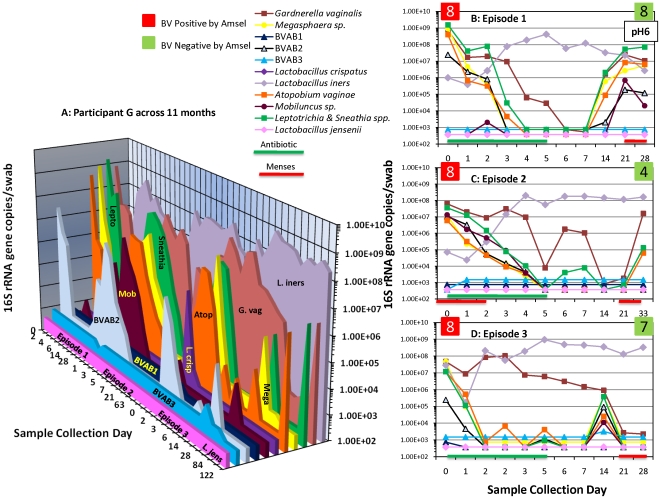
Bacterial fluctuations in a participant with recurrent BV. Figure 4A depicts dynamic patterns of BV bacteria in Participant G across a period spanning 11 months. She was diagnosed with BV by Amsel's and Nugent's criteria at entry (Episode 1, 4B), and was responsive initially to treatment. However, she had two more episodes subsequently (4C and 4D) and each time she responded to metronidazole treatment but had a return of BV-associated bacteria and went on to develop BV.

Three women (37.5%) in this study had at least ≥2 episodes of BV. Figure 4 shows recurrent BV in a representative participant with three episodes of BV. Participant G harbored several BV-associated bacteria and had a clinical diagnosis of BV at study entry (Episode 1, Figure 4B). With treatment, quantities of BV-associated bacteria declined below detection thresholds (except *G. vaginalis*). Subsequently, levels of BV-associated bacteria trended upwards reaching maximal levels during menses. At the 1-month clinic visit, Participant G was negative for BV by Amsel's criteria (pH 6, <20% clue cells, negative whiff test, normal discharge) and was not treated. Participant G returned to clinic 3 months later and was diagnosed with BV (Episode 2, Figure 4C). With metronidazole treatment, levels of BV-associated bacteria declined below detection thresholds and the participant was clinically BV negative at 1 month. However, she returned again in 3 months and was diagnosed with the third episode of BV (Episode 3, Figure 4D). With her third course of metronidazole, participant G had a similar response as previously observed, wherein there was initial decline of BV-associated bacteria and the participant was clinically negative for BV at 1 month. However, two months later quantities of *G. vaginalis*, *Leptotrichia/Sneathia* spp. and *A. vaginae* increased but she did not return to clinic for further examination. In all three episodes studied, *L. iners* had an inverse relationship with the BV-associated bacteria. Participant G was never colonized with *L. jensenii* and was briefly colonized with *L. crispatus* through the sampling period.

## Discussion

In our study of frequent sampling of the vaginal microbiota, we found that the bacterial community is dynamic and changes rapidly. We sought to study changes in the levels of bacteria in the human vagina and assess their relationship with the common condition, bacterial vaginosis. Our first goal was to determine how stable the microbial community is in healthy women. As noted in previous cultivation-based studies, healthy women tended to be colonized with several *Lactobacillus* species, though *G. vaginalis* was also frequently detected, including in 70% of women without BV using PCR methods [12]. *G. vaginalis* concentrations increased substantially with menses in 81% of monitored menstrual cycles, and levels decreased with the end of menstruation. During these surges of *G. vaginalis*, *L. iners* levels also trended upwards while levels of the other two lactobacilli decreased. Schwebke *et al*. [14] have similarly reported increases in *Gardnerella/Bacteroides* morphotypes by Gram stain and reduced quantities of lactobacilli during menses. Our study enhances the observations made by these authors by measuring levels of specific bacterial species and provides the ability to distinguish among *Lactobacillus* species. In our study, all subjects (n = 14) classified as negative for BV by the Amsel diagnostic criteria on Day 0 had at least a low level of *G. vaginalis* on one of the days sampled. A longitudinal cultivation study reported the isolation of *G. vaginalis* in at least one time point in the menstrual cycle in all subjects, though the number of subjects examined was small (n = 7) [15]. These findings raise the question of the role of the normal fluctuations of *G. vaginalis* in the development of abnormal flora associated with BV.

The growth of *G. vaginalis* may be tied to the availability of iron. Iron is an essential growth factor for most bacteria and the acquisition of iron enhances the replication of many pathogens [16], [17], [18]. There is limited free iron in the human body as much of it is sequestered in compounds such as hemoglobin, the iron-containing metalloprotein in erythrocytes, and lactoferrin, present in mucosal tissues. One mechanism for acquiring iron is to lyse host cells such as erythrocytes with a cytolysin thereby liberating intracellular iron stores. *G. vaginalis* produces a toxin, vaginolysin, a member of the cholesterol-dependent cytolysin family of toxins [19], [20]. Experiments examining the growth of *G. vaginalis* have shown that this bacterium cannot grow in iron-limiting conditions, but can use iron sources such as hemoglobin for growth and can produce siderophores suggesting a well adapted ability to harvest iron from the environment [21]. Our observation that surges in *G. vaginalis* coincide with menses (and therefore vaginal blood) is consistent with this hypothesis. As to the role of *G. vaginalis* in BV, we hypothesize that *G. vaginalis* may function as a facilitator to enhance acquisition of other BV-associated bacteria that are also characteristic of this condition.

In healthy women, concentrations of *L. iners*, when present, tended to increase along with levels of *G. vaginalis* during menses (Figures 1 & 2). It is noteworthy that both *G. vaginalis* and *L. iners* are easily cultivated on blood agar medium. As noted with Participant G on multiple occasions, concentrations of *L. iners* tended to increase with antibiotic treatment for BV, suggesting that this bacterium may fill the niche vacated by the loss of BV-associated bacteria.

Our second goal was to determine if BV-associated bacteria were eradicated with antibiotic treatment in women with recurrent BV, and to assess the time to eradication. We found that quantities of BV-associated bacteria decreased rapidly with intravaginal metronidazole, evidencing multi-log declines on a daily basis, though the slope of this decline varied (Figures 3 & 4). This observation supports the hypothesis that recurrent BV is not a result of initial antibiotic failure, but rather is associated with the reappearance of BV-associated bacteria after completion of antibiotic therapy. We are able to detect as few as 2.5 gene copies per qPCR reaction translating to 375 16S rRNA copies per swab. Hence, if the bacteria were present below these concentrations, we could not have detected them. Understanding how women with recurrent BV reacquire the BV bacteria is critical for prevention efforts.

This study has some limitations. First, there were a small number of subjects in this study. Longitudinal studies collecting many samples require highly motivated participants. Some investigators have taken the approach of processing limited numbers of samples from many women [9], [22], [23], [24], [25]. Our approach was to process large numbers of samples from a relatively small number of women in order to better explore vaginal bacterial dynamics. Second, we applied 11 bacterial assays; but this does not represent all bacterial taxa associated with the vagina. Complementary molecular technologies such as broad-range PCR, cloning and sequencing, or pyrosequencing may help overcome this limitation, though these approaches are not quantitative. Third, although daily qPCR data were obtained from the subjects, this was not correlated with daily clinical data such as exams since the swabs were mostly self-collected. Fourth, all subjects were from a single clinic and may not be broadly representative of reproductive age women with and without BV. Fifth, patterns of bacterial fluctuations were statistically examined in retrospect; hence p-values and 95% CIs arise from exploratory analyses and serve as a basis for future hypothesis-driven research.

In conclusion, the vagina is a dynamic microbial ecosystem supporting a changing and diverse bacterial population, both in healthy women and women with BV. Lactobacilli predominantly occur in healthy women, although their *Lactobacillus* species profiles vary. Subjects with BV have many fastidious BV-associated bacteria that respond well to the 5-day metronidazole treatment regimen, suggesting that these bacteria are susceptible to metronidazole or are dependent on other bacteria that are eradicated with antibiotic treatment. Recurrence of BV is associated with reappearance of BV-associated bacteria suggesting re-infection or resurgence from an endogenous reservoir. The rate of decrease of the BV bacteria with antibiotic treatment varies, suggesting that longer antibiotic treatments may be warranted in some women.
